# Application of a conceptual model to predict physical activity identity among Canadian adults

**DOI:** 10.1111/bjhp.70073

**Published:** 2026-04-10

**Authors:** Michael K. Smith, Alfred S. Y. Lee, Ryan E. Rhodes

**Affiliations:** ^1^ University of Victoria Victoria British Columbia Canada; ^2^ University College Dublin Dublin Ireland

**Keywords:** behaviour change, exercise identity, identity, physical activity

## Abstract

**Background:**

Evidence supports the benefits of physical activity (PA); however, many adults do not achieve recommended PA levels. Identity theories have been used to understand PA, but the antecedents of PA identity remain less clear.

**Purpose:**

This study presents an exploratory test of a conceptual model predicting PA identity strength using candidate antecedents identified in a recent narrative review.

**Methods:**

A three‐week prospective survey was administered to 570 Canadian adults. Time 1 recorded weekly self‐reported minutes of moderate‐to‐vigorous physical activity (MVPA), self‐regulation domains and five candidate antecedents—social relatedness, personal investment, perceived capability, PA alignment and priority. PA identity was measured 3 weeks later (Time 2). Using a structural equation modelling approach, we explored the proposed pattern of direct and indirect paths.

**Results:**

The model fit the data adequately, χ^2^ = 795.92, *df* = 324, CFI = .95, TLI = .95, RMSEA = .05 (90% CI = .05–.06) and SRMR = .07. Significant direct paths to Time 2 identity were observed for Time 1 MVPA, reactive regulation, self‐monitoring, relatedness, perceived capability, alignment and priority (*β* = .11–.34, *p*s ≤ .04). Indirect paths from Time 1 relatedness, personal investment and priority to Time 2 identity—operating via Time 1 MVPA and self‐regulation strategies (especially reactive regulation and self‐monitoring)—were also significant (*β* = .09–.18, *p*s ≤ .01).

**Conclusion:**

PA identity may have multivariate inputs. These findings provide preliminary evidence for the proposed conceptual model and experimental work is needed to determine whether modifying these inputs changes PA identity.

**Statement of Contribution**

**What is already known on this subject?**

Regular moderate to vigorous physical activity (MVPA) is essential for optimizing health outcomes however many individuals do participate regularly in MVPA (U.S. Department of Health and Human Services, 2018; Ross et al., 2020; World Health Organization, 2021). Physical activity (PA) identity is a reliable correlate of PA (Alfrey et al., 2025; Rhodes et al., 2016; Rhodes & Rebar, 2018; Strachan et al., 2011; Strachan & Brawley, 2008). Although candidate antecedents of PA identity have been proposed, more evidence is needed on how these inputs may be structurally aligned (Strachan, Kullman, & Rhodes, 2025).

**What does this study add?**

- This study provides an exploratory test of a conceptual model of PA identity using candidate antecedents identified in prior theory and review work.
- The findings identify theoretically consistent direct and indirect paths linking MVPA, self‐regulation and candidate antecedents with later PA identity.
- The results offer preliminary targets for future experimental research aimed at strengthening PA identity and supporting long‐term PA.

## INTRODUCTION

The link between regular moderate‐to‐vigorous intensity physical activity (MVPA) and positive health outcomes is well established. An extensive body of evidence exists that supports the benefits of MVPA as a primary and secondary means to prevent a range of chronic diseases, including coronary heart disease, stroke, Type 2 diabetes and hypertension (U.S. Department of Health and Human Services, 2018; Ross et al., 2020). Subsequently, physical inactivity is one of the leading risk factors for noncommunicable diseases, being associated with 4–5 million deaths annually (World Health Organization, 2021). Regular MVPA also has utility for improving cognitive function, reducing anxiety and depression and optimizing psychological health (U.S. Department of Health and Human Services, 2018; Ross et al., 2020). However, despite the myriad of well‐known health benefits of MVPA, many people do not participate regularly in MVPA. Approximately one in three adults self‐report that they fail to achieve recommended levels of MVPA, and this number is considerably larger for high‐income countries (Strain et al., 2024). For example, in Canada, direct assessment of MVPA shows that at least four of five adults do not meet these public health recommendations for the behaviour (Clarke et al., 2019).

Bettering our understanding of the factors driving adult MVPA participation is critical to creating programs aimed towards promoting active lifestyles. In particular, an increasing focus for health promoters is to understand factors that may be instrumental in maintaining the behaviour after the initial start of participation (Dunton et al., 2022; Rhodes & Sui, 2021). Many factors, which are socioecological in breadth, may be key in understanding ongoing MVPA participation (see (Kwasnicka et al., 2016) for a review). MVPA identity is one such factor that may be a keystone regulator in maintaining health behaviours like MVPA (Caldwell et al., 2018; Dunton et al., 2022; Epiphaniou & Ogden, 2010; Kwasnicka et al., 2016; Rhodes, Rebar, & Strachan, 2025; Rhodes & Sui, 2021).

*Identity*, often conceptualized as role identity in the PA domain, involves self‐categorization in a given role as part of a multi‐dimensional, hierarchically organized, self‐concept (Burke & Stets, 2023). This role‐based conception of identity is distinct from, yet closely related to, social identity theory and self‐categorization theory (Turner & Reynolds, 2012). Both role and social identity concepts are based on the same cognitive system, but functionally operate on different levels of categorization. Social identity reflects one's membership in a social group and is associated with norms at a higher, group‐based level, whereas role identity reflects one's position and obligations within specific intrapersonal or institutional roles (Stryker & Burke, 2000; Turner & Reynolds, 2012). Although functioning at different levels of self‐categorization, both guide behaviour through internalized expectations, with the higher social level constructs influencing an individual's interpersonal role obligation (how one's role identity is understood and performed) within the social structures (Stryker & Burke, 2000; Turner & Reynolds, 2012).

Identities are theorized to serve as personal standards of behaviour (Stryker & Burke, 2000) that support a dynamic, self‐regulating control system (Burke, 2006) guided by two interacting systems: reflective and automatic. The automatic system is driven by cues and stimuli, while the reflective system is influenced by an individual's goals/beliefs, and further, identity (Rhodes, Rebar, & Strachan, 2025; Strack & Deutsch, 2004). Specifically, the identity standard acts as a comparator to one's observed behaviour, and is activated by relevant situational cues (e.g., social, environmental, performance). Ultimately, cues (internal or external) that foster alignment experiences serve to strengthen the role identity; discrepancies between the cue and identity standard challenge an identity (Burke & Stets, 2023). This challenge produces a sense of incongruence that serves to motivate the performance of identity‐consistent behavioural actions (Strachan et al., 2009; Stryker & Burke, 2000). In the context of PA behaviours, an individual who views being physically active as an important part of who they are is more likely to choose activities that reinforce that identity and avoid immediate impulses that do not align with that image. This model of behavioural regulation has been supported in a series of hypothetical vignettes (e.g., asking participants how they would feel abstaining from PA) studies by Strachan and colleagues (Rhodes & Rebar, 2018; Strachan et al., 2011; Strachan & Brawley, 2008).

The association between PA identity and behaviour is now well‐established. A meta‐analysis of 32 studies found an association of *r* = .44 (CI = .39–.48) between PA identity and behaviour (Rhodes et al., 2016), suggesting that identity is a reliable correlate of PA. This effect has also been shown to persist even when accounting for additional moderators including intention, self‐determined motivations and habits (Alfrey et al., 2025). However, despite the preliminary supportive evidence for the importance of PA identity and behaviour, less is known about how to effectively intervene in PA identity. For example, a recent meta‐analysis of 40 studies featuring intervention effects on PA identity showed that although PA identity can change from intervention (*g* = .18; 95% CI = .11 to .24), follow‐up analyses could not identify any behaviour change techniques underlying differences in effect sizes (Rhodes et al., 2024).

At present, this lack of unifying understanding in promoting PA identity may stem from underdeveloped theoretical research on identity antecedents. Strachan and colleagues (Strachan, Kullman, & Rhodes, 2025) recently attempted to fill this theoretical gap with a critical narrative review of identity models of PA and health psychology. Models included: identity theory (Burke & Stets, 2023), the PA self‐definition model (Kendzierski & Morganstein, 2009), maintain identity transformation (Caldwell et al., 2018), multi‐process action control framework (Rhodes, 2021), PRIME theory (West, 2009), possible selves (Markus & Wurf, 1987) and self‐determination theory (Deci & Ryan, 1985). Using content analysis, they identified nine candidate antecedents of PA identity: (1) PA behaviour itself (repeated and consistent engagement in PA), (2) perceived ability (one's believed competence or capability at a specific task), (3) self‐regulation (how individuals manage their emotions and behaviours to achieve goals and avoid negative outcomes), (4) investment (self‐regulating, committing to, and investing behaviours in an identity), (5) rules/standards (clear criteria or standards around what it means for oneself to be a physically active person), (6) social attachment ties (close, supportive relationships formed through PA that foster belonging and relatedness), (7) alignment with goals or values (the reflection of one's beliefs and priorities), (8) social appraisals (identity‐relevant feedback, comparisons, or encouragement from others that affirm or challenge one's PA identity, such as social monitoring) and (9) imaginal experiences (mental imagery utilized to visualize one's self as a physical activity individual).

While Strachan (Strachan, Kullman, & Rhodes, 2025) highlights potential candidate inputs on identity, they recommend the need for evidence on how these inputs may be structurally aligned into a cohesive model, and testing of the relative contribution of these antecedents. The authors were also less certain of the proposed causal ordering of these constructions. Drawing from the candidate theories with identity, they highlighted that reflecting on past behavioural enactments may be critical to self‐categorization due to visible, self‐observational data for evaluating identity‐relevant patterns and thus, an important direct antecedent of PA identity (Bem, 1972; Kendzierski et al., 1998; Rhodes et al., 2017). Regulation behaviours are also frequently recalled when reflecting on PA identity (Strachan et al., 2017), supporting the conceptualization that these particular inputs and their enactment experiences may be direct antecedents of PA identity. All other potential PA identity inputs, as noted by Strachan et al. (Strachan, Kullman, & Rhodes, 2025), may have indirect effects through behavioural enactment and self‐regulation experiences or directly on PA identity or direct effects to the extent that these PA identity inputs represent unique determinants. Ultimately, evidence is needed to test how these potential inputs may interrelate when explaining PA identity.

**FIGURE 1:**
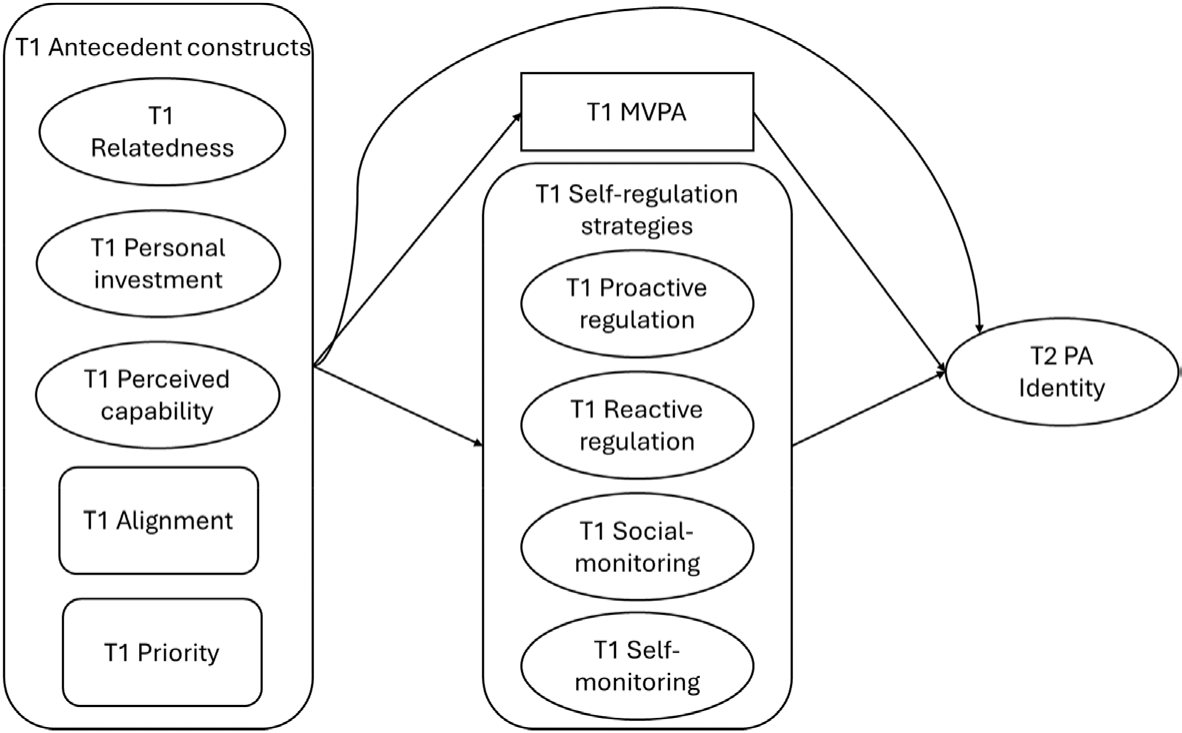
Conceptual structural equation model. The proposed structural equation model specifies paths from T1 antecedent constructs (relatedness, personal investment, perceived capability, alignment and priority) to T1 MVPA and T1 self‐regulation strategies (proactive regulation, reactive regulation, social monitoring and self‐monitoring), as well as paths from MVPA and self‐regulation strategies to T2 PA identity. Direct paths from the antecedent constructs to T2 PA identity were also specified. MVPA, moderate‐to‐vigorous physical activity; T1, baseline; T2, follow‐up; PA, physical activity.

The present study prospectively examined an integrated model of PA identity development. Guided by Strachan et al.'s (Strachan, Kullman, & Rhodes, 2025) narrative synthesis, baseline MVPA together with four self‐regulatory processes—proactive regulation, reactive regulation, social monitoring and self‐monitoring—were specified as proximal predictors of PA identity assessed 3 weeks later, whereas five additional proposed antecedents from Strachan et al. (relatedness as a proxy for attachment ties, personal investment, perceived capability, PA alignment and priority) were treated as more distal influences transmitted through MVPA and self‐regulatory processes (See Figure 1). Using an exploratory structural equation modelling approach, we examined the following hypotheses:

Hypothesis 1 Baseline MVPA was expected to have positive and direct effects on prospective PA identity.

Hypothesis 2 Each self‐regulation process was anticipated to exert a positive direct effect on later PA identity.

Hypothesis 3 Relatedness, personal investment, perceived capability, PA alignment and priority were expected to show positive indirect effects on PA identity through MVPA and the self‐regulating variable.

Hypothesis 4 Relatedness, personal investment, perceived capability, PA alignment and priority were expected to show positive direct effects on PA identity.

## METHODS

### Design and participants

The study featured a three‐week prospective, observational design using a third‐party market research platform with two measured time points. The baseline survey (T1) ran from 20 June to 2 July 2024. The second, follow‐up survey (T2) ran from 8 July to 12 August 2024. Canadian adults between the ages of 19–65 were sampled. All participants were English speakers living in Canada and self‐reporting no issues limiting their ability to perform physical activity. An a priori power analysis (α = .05, two‐tailed) indicated that a minimum of approximately *N* = 350 participants would provide ~80% power to detect small‐to‐moderate correlations (*r* ≈ .15) (Cohen, 1992). Participants were invited from the MARU market research platform, which has a database of approximately 120,000 Canadian panelists. The panel is representative of Statistics Canada's numbers in terms of various demographic variables (Government of Canada, S. C, 2001). All participants provided informed consent, and the study was approved by the appropriate research ethics boards.

### Procedures

Information about the study was distributed via the Maru group platform. Interested participants were then screened (19–65 years old) by Maru and eligible participants received a link to the study consent form and questionnaire. The study was conducted via SurveyMonkey and used an online design. The baseline and follow‐up surveys took approximately 20 and 10 min respectively, for a total of 30 min commitment for the entire study. Participants received a points‐based panel honorarium for each completed survey wave (paid separately at each wave): approximately CAD$1.30–$1.60 at baseline and CAD$1.50–$1.75 at follow‐up.

### Measures

#### Primary outcome: PA Identity

The primary outcome of the study was PA role identity measured via 3 items on a 5‐point scale from strongly disagree (1) to strongly agree (5) as adapted from Wilson and Muon (Wilson & Muon, 2008) from the exercise identity scale (Anderson & Cychosz, 1994). These items included ‘I consider myself someone who does regular physical activity’, ‘When I describe myself to others, I usually include my involvement in physical activity’, and ‘Others see me as someone who does physical activity regularly’. Measures of PA identity displayed acceptable internal consistency (α = .89).

#### Predictor measures

*MVPA* was measured using the Godin Leisure‐Time Questionnaire (Godin et al., 1986; Godin & Shephard, 1985), modified to include assessments of weekly PA frequency and duration. The multiplicative (frequency × duration) sum of moderate and vigorous intensity minutes was used as the estimate of weekly MVPA (Courneya et al., 2004). The Godin Leisure‐Time Questionnaire has been shown to be a valid and reliable measure of MVPA (Alotaibi et al., 2024; Godin & Shephard, 1985).

The self‐regulation concept was measured using the PA regulation scale (Rhodes & Lithopoulos, 2023) through 4 sub‐variables: *Proactive regulation, Reactive regulation, social monitoring* and *Self‐monitoring*, all measured on a 7‐point scale from strongly disagree (1) to strongly agree (7). *Proactive regulation* was tested via 4 questionnaire items (e.g., i. …when to be physically active; ii. …where to be physically active). *Reactive regulation* was tested via 4 items (e.g., i. When I am upset, I use strategies to feel better so I can be physically active; ii. When I am upset, I have ways of coping so I can focus on my physical activity plans). *Social monitoring* was tested via 3 items (e.g., i. There is someone who could provide feedback on my physical activity participation when needed; ii. I ask someone to remind me to be physically active). *Self‐monitoring* was tested via 3 items (e.g., i. I keep track of my physical activities; ii. I record my physical activities). All 4 sub‐variables displayed adequate internal consistency (Proactive regulation [α = .95], Reactive regulation [α = .92], social monitoring [α = .84], Self‐monitoring [α = .94]).

The social concept for the model was measured through PA *Relatedness*, which was assessed via 3 items on a 5‐point scale from strongly disagree (1) to strongly agree (5). Items were applied from Vlachopoulos et al. (Vlachopoulos et al., 2010). Example items included ‘My relationships with the people I exercise with are very friendly’ and ‘I feel I have excellent communication with the people I exercise with’. ‘Measures of relatedness displayed adequate internal consistency’ (α = .98).

The investment concept for the model was measured through *Personal investment* (Wilson et al., 2004) tested via 3 items (‘Invested lot of effort’; ‘Invested lot of energy’: ‘Invested lot of time’) on a 5‐point scale from strongly disagree (1) to strongly agree (5), based on an initial prompt (circle the response that best describes how you usually feel about exercise). Personal investment displayed adequate internal consistency (α = .93).

The perceived ability concept was measured through *Perceived Capability over PA* (Lithopoulos et al., 2023), assessed via 3 items on a 5‐point scale from strongly disagree (1) to strongly agree (5). Example items included ‘I possess the skills to do regular physical activity over the next month if I wanted to’, and ‘I am confident that I am capable of engaging in regular physical activity if I had to’. Perceived capability displayed adequate internal consistency (α = .90).

The alignment with goals/values concept was measured *through PA alignment with other values*, which was tested via 1 study‐created item (Regular physical activity aligns with the other values in my life) on a five‐point scale from strongly disagree (1) to strongly agree (5).

Priority, adapted from constructs of goal priority (Conner et al., 2016), was measured using a single item (e.g., Regular PA is…) rated on a seven‐point scale ranging from high on my priority list (1) to low on my priority list (7). Scores were reverse‐coded so that higher values reflected a higher priority given to PA.

### Demographics

Baseline measures collected via the first online survey included self‐reports of general demographic variables, including age, gender, ethnicity, education level/status, marital status, income, employment status, height and weight. The questionnaire also included health‐related screening questions including height/weight, smoking status, medications, self‐rated health and chronic disease status.

### Data analysis plan

Descriptive statistics, including means, standard deviations, reliability coefficients and correlations, were calculated for all study variables. A dropout analysis was conducted using independent sample *t*‐tests to examine whether participants who completed the post‐test survey differed from those who did not in terms of demographic characteristics (i.e., gender, age, education level, income, employment status and health profile) and baseline study variables (i.e., relatedness, personal investment, perceived capability, alignment, priority, MVPA and the four dimensions of the PA regulation scale). These analyses were conducted using SPSS v26 (IBM Corp, 2019).

To test the hypotheses, we used structural equation modelling to examine the proposed model (Figure 1). We tested a model where T1 exogenous variables (relatedness, personal investment, perceived capability, alignment and priority) predicted T2 PA identity both directly and indirectly via T1 MVPA and the four dimensions of PA regulation scale: proactive regulation, reactive regulation, social monitoring and self‐monitoring. In a planned sensitivity analysis, we re‐estimated the model, including Time 1 PA identity as a predictor of Time 2 PA identity, to examine whether associations with follow‐up identity were independent of baseline identity.

Model fit was assessed using conventional fit indices, with the following thresholds indicating acceptable fit: Comparative Fit Index (CFI) and Tucker–Lewis Index (TLI) values ≥.90 and root mean square error of approximation (RMSEA) and standardized root mean square residual (SRMR) values <.08 (Hu & Bentler, 1999). Analyses were conducted using all available cases; participants were not required to have complete data at all time points. Missing data was handled using the full information maximum likelihood (FIML) method, which retains participants with partial data by computing case‐wise likelihoods from their observed variables (Muthén & Muthén, 2017). All structural equation models were conducted using Mplus version 7.2 (Muthén & Muthén, 2017).

## RESULTS

### Preliminary statistics

A total of 570 participants (*M*_age_ = 48.51 ± 12.41, range = 19 to 65; female = 54.6%) responded to the survey invitation. General demographics and health information are reported in Table 1. Participants were primarily white (72.6%) and educated (71.0% held a college or university degree); however, the sample showed variability in employment (56.1% were employed full‐time) and income (30.7% reported an annual income of $100,000 or more). Participants also reported variability in the presence of chronic diseases, including high blood pressure (17.5%), high blood cholesterol (12.6%) and type 2 diabetes (6.5%). A small proportion of participants (9.6%) reported being smokers.

**TABLE 1:** Participants' demographic information.

Participants demographic profile	
Age in years (*SD*)	48.51 (12.41)
% Female	54.6
% Caucasian	72.6
% 4‐year college and above	71.0
% Income $100 k and above	30.7
% Full‐time employed	56.1
% Retired	20.0
% Children living at home	30.8
Health profile	
% Smoker	9.6
% Angina	.9
% Heart attack	.9
% Stroke	.4
% Type1 Diabetes	1.1
% Type2 Diabetes	6.5
% Gestational Diabetes	.5
% Cancer	3.2
% High blood pressure	17.5
% High blood cholesterol	12.6
Self‐reported health	
% Poor	3.4
% Fair	20.4
% Good	42.2
% Very good	26.5
% Excellent	7.6

A total of 389 participants completed the follow‐up survey, yielding a 68.2% retention rate. Results from Little's Missing Completely at Random test (Little & Rubin, 2019) indicated that missing data were not systematically patterned, χ^2^ = 27.48, *df* = 29 and *p* = .55. According to the dropout analysis, there were no significant differences in baseline demographic characteristics or study variables between participants who completed the follow‐up survey and those who did not, *t*(125) = −2.01 to 1.71 and *p*s = .05 to .96, except for age and type 2 diabetes status. Participants who dropped out were significantly younger than those who remained in the study, *t*(125) = −2.57, *p* = .01, and a greater proportion had type 2 diabetes, *t*(125) = −2.12 and *p* = .04. Descriptive statistics, including mean, standard deviations, reliability coefficients, skewness, kurtosis and correlations of study variables are presented in Table 2. All the study variables were positively and significantly correlated, *r* = .10 to .71 and *p*s < .01. Additionally, all variables fell within acceptable normality thresholds, with skewness ranging from −.89 to .68 and kurtosis from −1.59 to 1.27.

**TABLE 2:** Zero‐order correlations and descriptive statistics of the study variables (*N* = 570).

Variables	1	2	3	4	5	6	7	8	9	10	11
1. T1 Relatedness	1										
2. T1 Personal investment	.31**	1									
3. T1 Perceived capability	.15**	.40**	1								
4. T1 Alignment	.34**	.61**	.54**	1							
5. T1 Priority	.28**	.58**	.47**	.64**	1						
6. T1 MVPA	.25**	.46**	.35**	.40**	.50**	1					
7. T1 Proactive regulation	.34**	.56**	.34**	.48**	.53**	.39**	1				
8. T1 Reactive regulation	.42**	.58**	.36**	.58**	.60**	.46**	.64**	1			
9. T1 Social monitoring	.44**	.29**	.10*	.21**	.17**	.14**	.34**	.45**	1		
10. T1 Self‐monitoring	.28**	.46**	.22**	.38**	.41**	.35**	.49**	.51**	.35**	1	
11. T2 Identity	.44**	.62**	.44**	.63**	.65**	.55**	.54**	.71**	.36**	.55**	1
Mean	2.44	3.36	4.21	3.68	4.56	3.36	4.46	4.23	3.74	3.96	3.00
SD	1.92	.98	.73	.95	1.77	3.31	1.71	1.49	1.56	1.87	1.2
Cronbach's alpha	.98	.93	.90	N/A	N/A	N/A	.95	.92	.84	.94	.89
Skewness	−.28	−.61	−.89	−.77	−.37	.68	−.59	−.43	−.09	−.13	−.21
Kurtosis	−1.59	.12	1.27	.59	−.81	−.42	−.58	−.43	−.84	−1.17	−.87

Abbreviations: MVPA, moderate‐to‐vigorous physical activity; T1, baseline; T2, follow‐up.

*p < .05, **p < .01.

### Testing the conceptual model of PA identity

**FIGURE 2:**
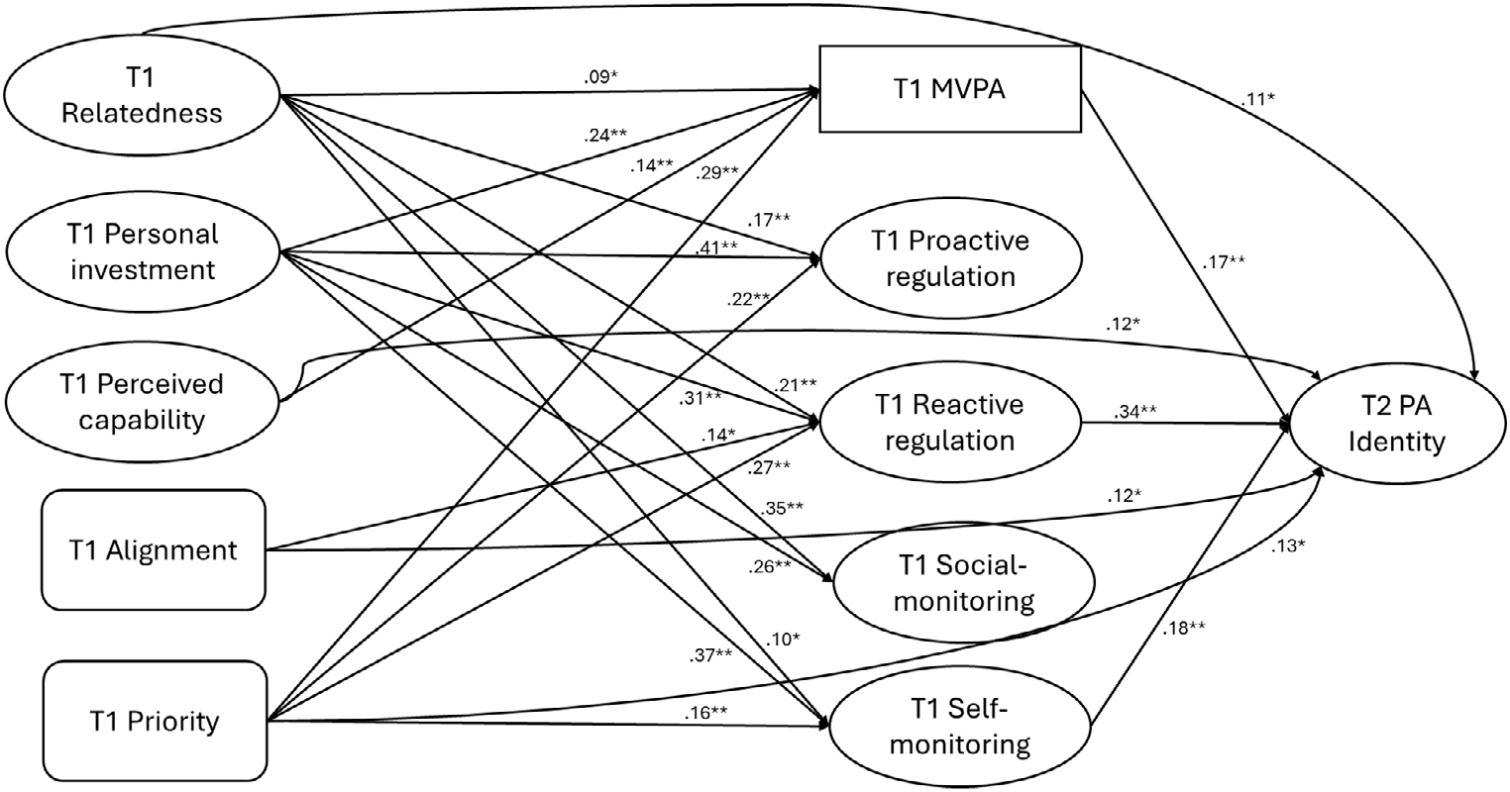
Structural equation model results with standardized estimates. Values represent standardized regression coefficients (β). For readability, only statistically significant structural paths are displayed; non‐significant paths were estimated but are not shown. *p < .05. **p < .01.

The proposed conceptual model displayed good fit to the data, χ2 = 795.92, *df* = 324, CFI = .95, TLI = .95, RMSEA = .05 (90% CI = .05–.06) and SRMR = .07 (see Figure 2). Among the exogenous predictors, relatedness and personal investment were positively associated with MVPA and all four self‐regulation strategies (*β*s = .09 to .41, *p*s < .001 to .02). Priority significantly correlated with MVPA and all four self‐regulation strategies (*β*s = .16 to .29, *p*s < .001 to .01), except social monitoring (*β* = −.03, *p* = .52). Perceived capability was positively associated with MVPA (*β* = .14, *p* = .01) but showed no significant associations with the four self‐regulation strategies (*β*s = −.04 to .06, *p*s = .07 to .72). Alignment did not significantly connect with MVPA and self‐regulation strategies (*β*s = −.04 to .10, *p*s = .32 to .69), except for reactive regulation (*β* = .14, *p* = .02). Regarding the predictors of subsequent PA identity, MVPA (*β* = .17, *p* < .01), reactive regulation (*β* = .34, *p* < .01) and self‐monitoring (*β* = .18, *p* < .01) showed significant direct associations with T2 PA identity. Additionally, relatedness (*β* = .11, *p* = .01), perceived capability (*β* = .12, *p* = .04), alignment (*β* = .12, *p* = .04) and priority (*β* = .13, *p* = .04) exhibited significant direct effects on follow‐up PA identity. However, personal investment (*β* = .08, *p* = .20), proactive regulation (*β* = −.06, *p* = .38) and social monitoring (*β* = −.01, *p* = .86) did not significantly associate with T2 PA identity. In Analysis S1, we report a baseline‐adjusted sensitivity model in which T2 PA identity is regressed on T1 PA identity. Time 1 PA identity strongly predicted Time 2 PA identity (*β* = .78, *p* < .001). After adjusting for baseline identity, MVPA (*β* = .09, *p* = .02), reactive regulation (*β* = .16, *p* = .01) and self‐monitoring (*β* = .11, *p* = .01) remained significantly associated with Time 2 identity, whereas proactive regulation (*β* = −.09, *p* = .06) and social monitoring (*β* = .01, *p* = .87) were not. Direct paths from the T1 antecedent constructs to T2 identity were attenuated and no longer statistically significant once baseline identity was included. These results suggest that, over this three‐week interval, most of the variance in follow‐up identity is accounted for by pre‐existing identity, as would be expected for a relatively stable construct; however, current behaviour and specific self‐regulation processes still show small additional associations with Time 2 identity after accounting for baseline identity.

In the primary model, the analysis of indirect effects (see Table 3) revealed that relatedness, personal investment, and priority had significant indirect effects on T2 PA identity via MVPA and/or the self‐regulation strategies. Specifically, relatedness demonstrated a significant indirect effect on follow‐up PA identity (*β* = .09, *p* = .01), primarily via MVPA (*β* = .02, *p* = .04) and reactive regulation (*β* = .07, *p* = .01). Personal investment exhibited the strongest indirect association (*β* = .18, *p* < .01), with its effects being indirectly associated with MVPA (*β* = .04, *p* = .01), reactive regulation (*β* = .10, *p* = .01) and self‐monitoring (*β* = .07, *p* = .01). Similarly, priority had a significant indirect effect on follow‐up PA identity (*β* = .15, *p* < .01), operating through MVPA (*β* = .05, *p* = .01), reactive regulation (*β* = .09, *p* = .01) and self‐monitoring (*β* = .03, *p* = .03). In contrast, perceived capability (*β* = .02, *p* = .50) and alignment (*β* = .08, *p* = .09) did not demonstrate significant indirect effects on follow‐up PA identity. When considering total effects (i.e., the combined direct and indirect effects), all exogenous variables significantly contributed to the subsequent PA identity, *β*s = .14 to .20, *p*s < .001 to .02.

**TABLE 3:** Results from the structural equation modelling with indirect paths.

Paths	Significant indirect effects (via)	Total indirect effects	Direct effects	Total effects
T1 Relatedness→ T2 Identity	T1 MVPA: *β* = .02* T1 Reactive regulation: *β* = .07**	.09**	.11*	.20**
T1 Personal investment → T2 Identity	T1 MVPA: *β* = .04** T1 Reactive regulation: *β* = .10** T1 Self‐monitoring: *β* = .07**	.18**	.09	.27**
T1 Perceived capability → T2 Identity	T1 MVPA: *β* = .02*	.02	.12*	.14*
T1 Alignment → T2 Identity	T1 Reactive regulation: *β* = .05*	.05	.12*	.17**
T1 Priority → T2 Identity	T1 MVPA: *β* = .05** T1 Reactive regulation: *β* = .09** T1 Self‐monitoring: *β* = .03*	.15**	.13*	.28**

*Note*: All effects are reported as standardized coefficients (*β*).

Abbreviations: MVPA, moderate‐to‐vigorous physical activity; T1, baseline; T2, follow‐up.

*p < .05, **p < .01.

## DISCUSSION

PA identity has an established positive relationship with PA behaviour (Rhodes et al., 2016; Rhodes, Rebar, & Strachan, 2025); however, less is known about how to effectively intervene upon PA identity (Rhodes et al., 2024). The objective of the current research was to conduct an exploratory test of a conceptual model of specifically identified candidate antecedents of PA identity based on a narrative review of PA and health psychology theories that include identity as an output (Strachan, Kullman, & Rhodes, 2025). In this exploratory structural equation modelling analysis, the conceptual model showed multiple significant direct and indirect paths to PA identity and demonstrated acceptable psychometric performance. Together, these antecedents explained 73% of the variance in follow‐up PA identity, underscoring the framework's explanatory power.

Commensurate with our first hypothesis, MVPA showed a small but significant (Cohen, 1992) positive direct effect on PA identity. This finding is aligned with theorizing that a primary determinant of self‐categorization includes reflection upon past behaviour (Bem, 1972; Kendzierski et al., 1998; Rhodes, 2017) and is consistent with theories of self‐perception (Bem, 1972) and self‐verification (Swann et al., 2003), which view behavioural enactment as a form of PA identity reinforcement (Strachan, Kullman, & Rhodes, 2025). Specifically, engaging in PA may provide direct stimulation to one's perception as a physically active individual and thus informs their PA identity development (Strachan, Kullman, & Rhodes, 2025). Interestingly, the finding also supports the previous conjecture that PA identity may be a maintenance‐level construct because behavioural performance experiences are a pre‐requisite input (Burke, 2006; Rhodes, Wierts, et al., 2025) and PA identity formation is likely to develop over time from behavioural initiation (Spruijt‐Metz et al., 2015). These findings also support previous work on the role of identity and maintained behavioural change, specifically in the context of habit formation and seeking identity‐aligned behavioral opportunities (Cushan‐Kain et al., 2022; Rhodes et al., 2022).

MVPA participation may also act as a proxy representative of other PA‐relevant behaviors (e.g., talking about exercise, wearing PA‐related clothing) thought to strengthen PA identity (Burke & Stets, 2023; Rhodes, Rebar, & Strachan, 2025). Future research may benefit by discerning the relative importance of the behavioural enactment of PA from these aforementioned PA adjacent activities in predicting PA identity. From an applied perspective, these PA adjacent activities may also offer practitioners more strategic recommendations to apply in interventions than just promoting MVPA, such as the relationship between Instagram use (of PA participation images) and PA identity explored by Liu et al. (Liu et al., 2021). Additionally, encouraging individuals to reflect on their PA behaviours and frame each instance of PA behaviour as evidence that they are a physically active individual may help further strengthen self‐categorization (Kendzierski & Morganstein, 2009).

Similar to the support of our first hypothesis, we also observed direct effects of key self‐regulation domains (reactive regulation and self‐monitoring) on PA identity. Reactive regulation, which had a medium‐sized effect on PA identity in our model, represents the intrapsychic tactics people use to focus on action in the moment, typically embodying emotion regulation and adjacent affect regulation approaches (Braver, 2012; Duckworth et al., 2016; Gross, 2014; Koole et al., 2023; Rhodes, Barton, et al., 2025). Self‐monitoring, which had a medium‐sized effect on PA identity in our model, is a behavioural regulation process that features ongoing awareness and monitoring of behaviour (Inzlicht et al., 2021). Both self‐regulation domains have been linked to PA through behavioural interventions (Knittle et al., 2018; Michie et al., 2009; Rhodes, Barton, et al., 2025). In terms of the link between reactive regulation and PA identity, affect‐based behavioural control has previously been linked to PA identity strength (Strachan et al., 2009, 2011; Strachan & Brawley, 2008). This is likely a reciprocal relationship stemming from success experiences in reactive regulation that result in engaging PA, and thus strengthen PA identity (Rhodes, Barton, et al., 2025). Relatedly, the concept of ‘psychological flexibility’ in Acceptance and Commitment Therapy emphasizes on present‐moment awareness and values‐aligned action maps directly onto reactive regulation and has been shown to facilitate health‐behaviour change (Pears & Sutton, 2021; Zhang et al., 2018). Accordingly, interventions that cultivate psychological flexibility may provide a promising route for enhancing reactive regulation and, in turn, PA identity formation. From a practical perspective, the results support the cultivation of reactive regulation tactics to promote PA identity and behaviour (Strachan, Kullman, Dobrovolskyi, et al., 2025).

Additionally, in terms of the link between self‐monitoring and PA identity, individuals who are actively aware of their behaviour may enable self‐categorization compared to those with lower behavioural awareness. Aligned with the similar rationale to how behaviour influences PA identity (Bem, 1972; Kendzierski et al., 1998; Rhodes et al., 2017) we speculate that self‐monitoring may specifically create a heightened opportunity to reflect on the performance, time, and effort of PA. From a practical perspective, ongoing self‐monitoring may be beneficial to behaviour change through the strengthening of PA identity (Knittle et al., 2018; Michie et al., 2009).

It was interesting to note that proactive regulation and social monitoring did not have significant independent effects on PA identity within the model. Both self‐regulation components, however, did have medium‐ to large‐sized bivariate correlations with PA identity (see Table 2), thus supporting an overarching relationship between PA identity and self‐regulation. Thus, the findings are in line with (Strachan, Kullman, & Rhodes, 2025) suggested inputs for self‐regulation to PA identity. The results of our conceptual model merely suggest that reactive regulation and self‐monitoring may be the critical independent self‐regulation contributors to PA identity. Sustained testing of multiple components of self‐regulation is recommended; however, to gain clarity on which properties of self‐regulation may be usefully targeted for interventions.

Also of interest in our analysis were the antecedent variables that did not have a significant relationship with PA identity (proactive regulation, social monitoring and personal investment) independent of the other variables in the model. As previously discussed, identity formation is strongly based upon the ability to evaluate identity‐relevant patterns that will then inform future behaviours to align with that identity standard (Bem, 1972; Kendzierski et al., 1998; Rhodes et al., 2017). Our null findings may suggest that proactive regulatory strategies are enacted prior to this evaluative process and therefore do not readily prompt reflection on identity‐relevant patterns. Regarding social monitoring, this is a somewhat surprising finding within our analysis, given the evidence suggesting the importance of social comparisons in building PA identity (Strachan, Kullman, & Rhodes, 2025). This finding may be due to the relevance of relatedness within a PA environment. If an individual's relationship is not grounded in activity values/norms, the social context lacks the symbolic significance required to embed ‘being active’ into their self‐concept (Burke, 2025). Finally, personal investment revolves around an individual's inputs (time, energy, money) and may signify a level of external investment that does not necessarily translate into self‐defining characteristics (Strachan, Kullman, & Rhodes, 2025). Although these variables were not significant predictors in the present study, clarifying the conditions under which they may support or hinder PA identity remains an important avenue for future research on health‐related behaviours.

Within our indirect model of PA identity antecedents, MVPA and reactive regulation also stood out as key indirect associations. Specifically, MVPA was indirectly associated with paths from relatedness, personal investment, perceived capability and priority to PA identity, whereas reactive regulation was indirectly associated with the effects of relatedness, personal investment, alignment with values/goals and priority. These findings support our hypothesis that several candidate inputs may be explained through behavioural and self‐regulatory effects. The prominence of MVPA in these indirect pathways is consistent with the notion that behavioural enactment reinforces PA identity (Strachan, Kullman, & Rhodes, 2025). In addition, the indirect pathways involving reactive regulation may reflect individual differences in managing internal affective states, consistent with emotion regulation and general affect regulation approaches (Pears & Sutton, 2021; Rhodes, Barton, et al., 2025; Zhang et al., 2018). Of particular theoretical interest in these indirect effects was the effect of personal investment, which Strachan et al. (Strachan, Kullman, & Rhodes, 2025) highlighted as likely having a direct effect on PA identity; our conceptual model showed that investment only had an indirect effect via MVPA, reactive regulation and self‐monitoring. Although investment has been strongly linked to exercise commitment (Wilson et al., 2004) and theorized as critical to self‐categorization (Kendzierski & Morganstein, 2009; Rhodes, 2017), these findings suggest that investing in PA behaviours through personal resources, energy and/or time may affect PA identity through behavioural enactment or regulation tactics.

Finally, our proposed PA identity model showed that relatedness, perceived ability, alignment and priority each made small, positive direct contributions to predicting follow‐up PA identity. It is important to note that these results were found after controlling for MVPA and self‐regulation, so there is reflection on self‐categorization beyond just behavioural/self‐regulatory experiences. Relatedness, described as the authentic association with significant others and the sense of belonging, is an aspect of the psychological need fulfilment within SDT and sits as a clear representation of attachment ties in Rhodes and Beauchamp's (Rhodes & Beauchamp, 2024) Social Dimensions of Health Framework. The effects of relatedness are facilitated primarily through the concept of integrated regulation, synonymous with PA identity, where individuals engage in behaviours because doing so is consistent with how they view themselves (Strachan, Kullman, & Rhodes, 2025) The application of social attachment ties constructs, such as relatedness, within a PA setting has been shown to be positively associated with increased physical activity (Lim et al., 2021; Rhodes et al., 2017; Vlachopoulos et al., 2010). Our results support the ongoing application of interventions to increase relatedness to promote PA identity directly and via MVPA behaviour and self‐regulation.

The concept of perceived ability is incorporated into many behavioural theory models, including Social Cognitive Theory (Bandura, 1986), the Theory of Planned Behaviour (Ajzen, 1991) and The Physical Activity Self‐definition Model (Morgan et al., 2021). PA alignment with individuals' goals and values is an overarching concept that revolves around minimizing identity conflict when engaging in PA behaviours. Techniques such as motivational interviewing focus on this by encouraging the alignment of PA behaviours with an existing PA identity to reduce the possibility of future identity conflict (Miller & Rollnick, 2023). Priority involved the level of significance that an individual places on certain goal‐oriented outcomes (Carron & Brawley, 2000). For many individuals, prioritizing PA behaviours involves aspects of sacrifice, discipline and organization of one's time (Carron & Brawley, 2000; Rhodes, 2017). Consistent with earlier work, our results indicate that prioritizing physical activity strengthens PA identity by supporting both automatic enactment of the behaviour and sustained, effortful engagement (Strachan et al., 2017). Our findings also suggest that environments fostering community, social connection, belonging and a sense of competence can facilitate the development of a strong PA identity (Strachan, Kullman, & Rhodes, 2025). Interventions that couple PA with participants' intrinsic goals and values and teach concrete strategies for prioritizing healthy PA behaviour can simultaneously raise activity levels and solidify a strong PA identity.

Despite the originality of this research and the population sample, several limitations and future directions of research should be considered when interpreting the current findings. The timeline between administering the T1 and T2 surveys was only 3 weeks, which limits our ability to draw conclusions about long‐term changes in PA identity, a construct generally conceptualized as relatively stable and resistant to short‐term change (Burke, 2006). We therefore treated T2 PA identity as the primary outcome and used T1 PA identity in a baseline‐adjusted sensitivity model to assess whether the T1 antecedent, behavioural, and self‐regulation variables were associated with follow‐up identity over and above baseline identity. Future studies with longer follow‐up and multiple assessments of PA identity are needed to more fully examine change processes. Another limitation was the use of self‐reported measures, specifically MVPA. Although the Godin Leisure‐Time Questionnaire has been shown to be a valid and reliable measure of MVPA (Alotaibi et al., 2024), self‐reported measures have been shown to generally overestimate PA levels and underestimate sedentary time when compared to device measures (Prince et al., 2020). The use of a direct measure of MVPA could extend upon the current findings.

As this is the first test exploring the antecedents of PA identity strength, further replication is needed to fully elucidate the relationship between PA identity and these predictive variables. Specifically, reciprocal relationships between measures were not assessed in the current model. Current theories do indicate potential reciprocal relationships between PA identity and multiple antecedent‐related variables (Burke & Stets, 2023; Rhodes & Sui, 2021). The use of longitudinal models in future research could help clarify these relationships. Future intervention studies could also test whether manipulating these candidate antecedents produces measurable changes in PA identity. Additionally, the ‘imaginal experiences’ candidate, identified by (Strachan, Kullman, & Rhodes, 2025), was not included in the current model, while the ‘social appraisal’ variable was narrowly defined as social monitoring in this model. Exploring these measures in more depth should be incorporated in future research. More specifically, due to the large body of research currently on social identity, this variable may need further investigation in its role in PA identity (Reynolds et al., 2020; Stevens et al., 2022).

Finally, the use of structural equation modelling using observational data entails several important limitations. First, although our model was specified a priori based on theory, unmeasured confounding may bias both direct and indirect effects, and alternative model specifications (e.g., reversed or reciprocal pathways) cannot be ruled out. Second, the observational nature of the data limits causal inference and does not allow us to definitively establish the temporal ordering among the antecedent variables, the self‐regulation strategies, MVPA and PA identity. Third, all variables were assessed via self‐report, which introduces measurement error that can distort estimates of direct and indirect effects (Cole & Preacher, 2014). These challenges, highlighted in prior methodological work on mediation and more recent guidance on estimating and interpreting indirect effects using observational data (Schuler et al., 2025), mean that our findings should be interpreted as preliminary evidence of theoretically consistent patterns of association rather than as confirmation of causal mechanisms. Future longitudinal and experimental work will be needed to more rigorously evaluate the proposed pathways.

In summary, through structural equation modelling analysis, a conceptual model of candidates' direct and indirect antecedents of PA identity was explored. MVPA was associated with PA identity, as were the self‐regulation components of reactive regulation and self‐monitoring. Relatedness, perceived ability, alignment and priority also displayed small independent effects on follow‐up PA identity. Further, MVPA and reactive regulation were indirectly associated with the effects of relatedness, personal investment and priority on PA identity. Collectively, these findings demonstrate the multifaceted pathways shaping PA identity and offer a foundation for interventions that bolster PA identity to sustain long‐term physical activity and healthier lifestyles.

## AUTHOR CONTRIBUTIONS

**Michael K. Smith:** Writing – original draft; writing – review and editing. **Alfred S. Y. Lee:** Writing – review and editing; formal analysis. **Ryan E. Rhodes:** Conceptualization; supervision; writing – review and editing.

## FUNDING INFORMATION

The authors have no financial relationships relevant to this article to disclose.

## CONFLICT OF INTEREST STATEMENT

The authors have no conflicts of interest relevant to this article to disclose.

## AI DISCLOSURE

No AI was used in the study or creation/writing of this manuscript.

## Supporting information


Data S1:

## Data Availability

The data that support the findings of this study are available on request from the corresponding author. The data are not publicly available due to privacy or ethical restrictions.
