# Supplementary material for: Application of a conceptual model to predict physical activity identity among Canadian adults
Source: Br J Health Psychol. 2026 Apr 10;31(2):e70073. doi: 10.1111/bjhp.70073 (PMC13067307; doi:10.1111/bjhp.70073)
Supplement: Supplementary file 1 — Data S1: [file BJHP-31-0-s001.docx]

**Supplementary Materials**

**Supplementary Analysis S1. Baseline-adjusted model controlling for T1 PA identity**

To address the concern that associations with follow-up PA identity may reflect baseline PA identity, we re-estimated the structural equation model including baseline (T1) PA identity as a predictor of follow-up (T2) PA identity. This baseline-adjusted model showed acceptable fit to the data, χ² = 1183.79, *df* = 405, CFI = .94, TLI = .93, RMSEA = .06 (90% CI = 0.05–0.06), SRMR = .07. Given the brief interval between waves, the main text presents the specified model as our primary analysis; we report this baseline-adjusted specification here as a sensitivity analysis controlling for T1 PA identity.

Overall, the pattern of associations between the antecedent constructs and the T1 behavioral and self-regulation variables was generally consistent with the primary model. Regarding predictors of follow-up PA identity, baseline PA identity was a strong predictor of follow-up PA identity (*β* = .78, *p* < .001). After adjusting for baseline PA identity, MVPA (*β* = .09, *p* = .02), reactive regulation (*β* = .16, *p* = .01), and self-monitoring (*β* = .11, *p* = .01) remained significantly associated with follow-up PA identity. In contrast, proactive regulation (*β* = -.09, *p* = .06) and social monitoring *(β* = .01, *p* = .87) were not significantly associated with follow-up PA identity.

After adjustment for baseline PA identity, the direct associations from the antecedent constructs to follow-up PA identity were not statistically significant (relatedness: *β* = -.03, *p* = .56; personal investment: *β* = -.01, *p* = .83; perceived capability: *β* = .04, *p* = .43; alignment: *β* = .07, *p* = .31; priority: *β* = -.05, *p* = .40). Collectively, these findings indicate that baseline PA identity accounts for a substantial proportion of variance in follow-up PA identity, while MVPA and select self-regulation variables retain unique associations with follow-up PA identity net of baseline identity.
